# Crucial Role of Elovl6 in Chondrocyte Growth and Differentiation during Growth Plate Development in Mice

**DOI:** 10.1371/journal.pone.0159375

**Published:** 2016-07-28

**Authors:** Manami Kikuchi, Masako Shimada, Takashi Matsuzaka, Kiyoaki Ishii, Yoshimi Nakagawa, Misa Takayanagi, Nobuhiro Yamada, Hitoshi Shimano

**Affiliations:** 1 Department of Internal Medicine (Endocrinology and Metabolism), Faculty of Medicine, University of Tsukuba, Ibaraki, Japan; 2 Graduate School of Nutritional Science, Sagami Women’s University, Kanagawa, Japan; 3 International Institute for Integrative Sleep Medicine, University of Tsukuba, Ibaraki, Japan; 4 AMED-CREST, Japan Agency for Medical Research and Development, Tokyo, Japan; Kyungpook National University School of Medicine, REPUBLIC OF KOREA

## Abstract

ELOVL family member 6, elongation of very long chain fatty acids (Elovl6) is a microsomal enzyme, which regulates the elongation of C12-16 saturated and monounsaturated fatty acids. Elovl6 has been shown to be associated with various pathophysiologies including insulin resistance, atherosclerosis, and non-alcoholic steatohepatitis. To investigate a potential role of Elovl6 during bone development, we here examined a skeletal phenotype of *Elovl6* knockout (*Elovl6*^*-/-*^) mice. The *Elovl6*^*-/-*^ skeleton was smaller than that of controls, but exhibited no obvious patterning defects. Histological analysis revealed a reduced length of proliferating and an elongated length of hypertrophic chondrocyte layer, and decreased trabecular bone in *Elovl6*^*-/-*^ mice compared with controls. These results were presumably due to a modest decrease in chondrocyte proliferation and accelerated differentiation of cells of the chondrocyte lineage. Consistent with the increased length of the hypertrophic chondrocyte layer in *Elovl6*^*-/-*^ mice, *Collagen10*α*1* was identified as one of the most affected genes by ablation of *Elovl6* in chondrocytes. Furthermore, this elevated expression of *Collagen10α1* of Elovl6-null chondrocytes was likely associated with increased levels of *Foxa2/a3* and *Mef2c* mRNA expression. Relative increases in protein levels of nuclear Foxa2 and cytoplasmic histone deacethylase 4/5/7 were also observed in *Elovl6* knockdown cells of the chondrocyte lineage. Collectively, our data suggest that Elovl6 plays a critical role for proper development of embryonic growth plate.

## Introduction

Metabolic syndrome is a combination of the medical disorders including obesity, hyperglycemia, insulin resistance, and dyslipidemia, and increases the risk for diabetes and cardiovascular diseases. It is estimated, for example, that approximately one thirds of the US population suffers from metabolic syndrome [1, 2]. Metabolic syndrome shows a significant correlation with development of skeletal diseases including orthopedic arthritis and osteoporosis [3, 4]. Thus, to identify molecular mechanisms underlying metabolic syndrome and its associated complications is an emerging need in medical investigation.

We have shown previously that changes in fatty acid composition in serum and various organs affect insulin sensitivity [5], formation of atherosclerotic lesions [6], and non-alcoholic steatohepatitis [7]. Elongation of very long-chain fatty acids family member 6 (Elovl6) belongs to a family of microsomal enzymes and regulates the elongation of saturated and monounsaturated fatty acids with 12, 14, and 16 carbons. Elovl6 is a major transcriptional target of sterol regulatory element-binding protein (SREBP) 1, a critical modulator for fatty acid-lipid biosynthesis and glucose metabolism [8, 9]. Clinically, genetic variations in the *Elovl6* gene have a significant association with insulin sensitivity in population-based studies [10, 11]. *Elovl6* is expressed ubiquitously and is unique in its role for *de novo* fatty acid biosynthesis [12]. Lack of *Elovl6* increases levels of palmitate (C16:0) and palmitoleate (C16:1 n-7); while, it decreases those of stearate (C18:0) and oleate (C18:1 n-9) in serum and various tissues. *Elovl6* knockout mice showed a marked resistant to diet-induced hyperinsulinemia, atherosclerosis, and steatohepatitis presumably due to the altered fatty acid composition in the liver and macrophages, respectively [5–7]. Interestingly, the birth rate of *Elovl6* knockout mice was lower, and the surviving mice were smaller in size and had reduced body weight [5]. We thus hypothesized that Elovl6 might be crucial during embryonic skeletal development. Very little has been known about an effect of fatty acid composition on growth plate development and chondrogenesis; a study showed that exposure to n-3 fatty acids accelerates bone growth by increasing chondrocyte proliferation and differentiation in growth plate [13]. Therefore, to test our hypothesis and identify a potential link between fatty acids and growth plate development, we performed basal skeletal analysis mainly on hind limb specimens isolated from control and *Elovl6* knockout perinatal mice in this study.

## Materials and Methods

### Animals

Mice lacking *Elovl6* in the genetic background of C57BL/6 were generated as described previously [5]. Mice with homozygous deletion of *Elovl6 (Elovl6*^-/-^) and age-matched control *Elovl6*^+/+^ littermates (*control*) were housed in a pathogen free barrier facility with a 14h light and 10h dark cycle. All animal husbandry and animal experiments in this study were carried out in strict accordance with the recommendations in the Guidelines for the proper implementation of animal experiments of the Science Council of Japan. The protocol was approved by the Committee on the Ethics of Animal Experiments of the University of Tsukuba (Permit Number: 15–190). All animal experiments potentially induced no more than momentary pain or distress caused by occasional routine injections and did not involve the use of pain relieving drugs. Postnatal animals were euthanized by CO_2_ inhalation with compressed CO_2_ gas. In case of embryo collection, pregnant mothers were euthanized by cervical dislocation after CO_2_ inhalation with compressed CO_2_ gas for a few minutes. Fifteen minutes after mothers' death, embryos were retrieved by Cesarian section.

### Fatty acid composition of costal cartilage and ATDC5 cells

Fatty acid composition of primary costal chondrocytes and ATDC5 cells was determined using gas chromatography (SRL Corp., Tokyo, Japan). Total lipids in primary costal chondrocytes and mouse chondrogenic cells, ATDC5 (RIKEN cell bank, Ibaraki, Japan), were extracted according to Bligh-Dyer method [14].

### Whole Mount Skeletal Staining

The whole mount skeletal staining was performed as described previously [15]. Briefly, embryonic day (E) 18.5 embryos and newborn mice were fixed in ethanol for 5 days and then in acetone for 2 days. Staining with Alizarin red S and Alcian blue was performed for 3 days at 37°C. After washing with distilled water, the skeleton was cleared with 1% KOH and taken through graded steps into 100% glycerol.

### Sample preparation and histological analyses

*Elovl6*^-/-^ and *control* littermates were sacrificed at E15.5, E17.5, E18.5, and birth. Tissues were fixed and stored as described previously [15]. In selected cases, hind limb samples were decalcified and paraffin blocks were prepared by standard histological procedure at the histology core in the University of Tsukuba. To examine bone morphology, some selected samples were stained with hematoxylin and eosin (H&E), periodic acid-Schiff (PAS), and Safranin O [16].

### *In situ* hybridization

*In situ* hybridization analysis was performed as described previously with slight modifications [16]. Complementary DNAs corresponding to mouse *Elovl6*, *collagen1α1* (*Col1*α*1*), *collagen2α1* (*Col2*α*1*), *collagen10α1* (*Col10α1*), and *tartrate-resistant acid phosphatase (TRAP)* were used to generate digoxigenin-labeled antisense riboprobes using Riboprobe systems from Promega (Madison, WI).

### Analysis of bromodeoxyuridine (BrdU) incorporation

For BrdU labeling, pregnant female mice with E18.5 embryos were injected intraperitoneally with 100μg BrdU and 12μg fluorodeoxyuridine per gram of body weight 2 hours before being sacrificed (Sigma-Aldrich, St. Louis, MO). To identify actively proliferating cells, nuclei that had incorporated BrdU were detected as described previously [15, 16].

### Microarray analysis

Total RNA was extracted from primary costal chondrocytes isolated from newborn *Elovl6*^-/-^ and control mice (n = 3, each group). Agilent Expression Microarray analysis was performed at Takara Bio INC. (Shiga, Japan). The data were analyzed using GeneSpring software (Tomy Digital Biology Co., Tokyo, Japan).

### Chondrocyte isolation and culture

Primary costal chondrocytes were isolated from newborn mice as described previously [16]. Briefly, the costochondral regions of newborn mice were carefully dissected, rinsed with phosphate-buffered saline, and digested in Dulbecco’s modified Eagle’s medium (DMEM) (Invitrogen Corp., Carlesbad, CA) supplemented with 0.25% type II collagenase (Worthington, Lackwood, NJ) for 2 hours at 37°C. The digested cells were cultured in DMEM containing 5% fetal bovine serum (FBS) (Sigma-Aldrich) and 1% penicillin-streptomycin (Invitrogen).

ATDC5 cells were cultured in DMEM-Ham’s F-12 (1:1) (DMEM-F12) (Invitrogen) supplemented with 5% FBS and 1% penicillin-streptomycin (growth medium). To induce chondrogenic differentiation, cells were incubated in a growth medium supplemented with insulin, transferrin, and selenium (ITS) (Sigma-Aldrich) (differentiation medium) [16]. In some cases, ATDC5 cells were incubated in a DMEM-F12 supplemented with 5% bovine serum albumin, 1% penicillin-streptomycin, ITS (modified differentiation medium) and either 50 μM palmitic acid or 50 μM stearic acid.

### Osteoclast differentiation from bone marrow cells

Bone marrow cells were isolated from 8- to 10-week-old control and *Elovl6*^*-/-*^ mice. Femurs and tibias were removed and dissected free of adherent soft tissues. The bone ends were cut, and the marrow cavity was flushed out with DMEM from one end of the bone using a sterile 21-gauge needle. The bone marrow was carefully dispersed by pipetting and incubated overnight in DMEM containing 10% FBS for 20h, and non-adherent cells were harvested and inoculated at 1×10^5^ cells/cm^2^ for 2 days in the presence of 10 ng/ml of M-CSF (R&D systems, Minneapolis, MN). Adherent cells were used as bone marrow macrophages (BMMs). To obtain osteoclastic cells, BMMs were further cultured with 100 ng/ml of RANKL (R&D systems) in the presence of 50 ng/ml of M-CSF for 5 days and cells were then stained with TRAP using TRAP/ALP stain kit (Wako Pure Chemicals, Osaka, Japan) [17]. TRAP-positive multinucleated cells with more than 3 nuclei were counted as osteoclasts.

### Real-time quantitative (q) PCR

Total RNA extraction was performed using Sepasol-RNA I Super G (Nacalai tesque, Kyoto, Japan). cDNA was synthesized with the PrimeScript RT Master kit (Takara Bio Company) and real-time qPCR analysis was performed using SYBR Premix Ex Taq II (Takara Bio). The results were normalized to cyclophilin expression. Primer sequences for real-time qPCR were as below [15, 16]. Col2a1, forward (fw)-CAGGTGCTAATGGCAATCCT; reverse (rev)-GGAGGACCATCAAGAC CAGA. Col10a1, fw-GAGGCCACGGAACAGACTCA; rev-CAGCGCCTTGAAGATAG CATT. Cyclophilin, fw-TGGCTCACAGTTCTTCATAACCA; rev-ATGACATCCTTCAG TGGCTTGTC. Elovl6-ver1, fw-ACAATGGACCTGTCAGCAAA; rev-GTACCAGTGCA GGAAGATCAGT. Elovl6-ver2, fw-GCCATCCTCTGTACCCGATA; rev-AACTGGCC CTTTCATCTGTG. Foxa2, fw-CCATCAGCCCCACAAAATG; rev-CCAAGCTGCCTG GCATG. Foxa3, fw-AACCCACTCAGCTCTCCCTAC; rev-CCTTTGCCATCTCTTTTC CAT. HDAC4, fw-CGCTATGACGATGGGAACTT; rev-CATCTGGGGCAAACTCATT T. HDAC5, fw-GTCGAAAGGATGGCACTGTT; rev-AGCCAGTAAAGCCGTTCTCA. HDAC7, fw-TTTCTACCAGGACCCCAGTG; rev-AAGCAGCCAGGTACTCAGGA. Igf-1, fw-GCTGCTGAAGCCATTCATTT; rev-TTGCTCTTAAGGAGGCCAAA. Lcn2, fw-CTGAATGGGTGGTGAGTGTG; rev-GGAGTGCTGGCCAAATAAGA. Mef2c, fw-ATCCCGATGCAGACGATTCAG; rev-AACAGCACACAATCTTTGCCT. PTH1R, fw-GAGTCTACATGTCTAGGGTCTA; rev-TAGTTGGCCCACGTCCTGT. Runx2, fw-CA GACCAGCAGCACTCCATA; rev-CAGCGTCAACACCATCATTC. Scd-1, fw-AGATC TCCAGTTCTTACACGACCAC; rev-CTTTCATTTCAGGACGCATGTCT. Scd-2, fw-T CCTGGCGCTTACTCAGCCA; rev-CATCTGCTCCCCAGTGGTG. Sox9, fw-CAAGC ACATTTTCCCTGGTT; rev-CGCTGGTATTCAGGGAGGTA.

### Gene silencing by short hairpin (sh) RNA

ATDC5 cells were infected with lentivirus encoding either control or *Elovl6* shRNA for stable gene silencing (shElovl6: TRCN0000345535; Sigma-Aldrich) [16]. Lentiviral vectors, pLKO.1-puro and TRC2-pLKO-puro, contain mammalian puromycin-resistant genes for selection of shRNA inserts in ATDC5 cells. Four clonal ATDC5 cell lines expressing *Elovl6* shRNA and three independent cell lines expressing *control* shRNA were established.

### Western blot analysis

Cytoplasmic and nuclear extracts were isolated using Paris kit (Thermo Fischer Scientific; Waltham, MA). Western blot analysis was performed as described previously [16]. Foxa2/HNF3β, laminA/C, α-tubulin, and histone deacethylase (HDAC) 4/5/7 antibodies were purchased from Cell Signaling Technology (Danvers, MA) and Santa Cruz Biotechnology (Dallas, Texas), respectively. The intensity of protein bands was semi-quantified using ImageJ 1.50i software [18].

### Statistical analysis

Data were expressed as the means ± standard error (SE). Statistical analysis was performed using the unpaired Student *t* test or one-way analysis of variance with Tukey’s or Dunnett’s multiple comparison test. *P* values less than 0.05 were accepted as significant.

## Results

### *Elovl6* was expressed in cells of the osteoblast, chondrocyte, and osteoclast lineages in mice

To examine spatial expression of *Elovl6* in bone and cartilage *in vivo*, *in situ* hybridization analysis of proximal tibias isolated from control newborn mice was performed using sense and anti-sense riboprobes for *Elovl6*. Relatively strong *Elovl6* mRNA expression was observed in periarticular and columnar chondrocyte layers and its weaker expression was likely detected in a hypertrophic chondrocyte layer and primary spongiosa (S1A Fig). To further test basal expression of *Elovl6* in cells of the osteoblast, chondrocyte, and osteoclast lineages, real-time qPCR analysis was performed on specimens of liver, bone, and various types of cells differentiated from bone marrow stromal cells of control mice. Cells of the osteoblast, chondrocyte, and osteoclast lineages showed 2- to 7-times higher *Elovl6* expression than liver (S1B Fig). These results suggest that *Elovl6* is ubiquitously expressed in cells of the osteoblast, chondrocyte, and osteoclast lineages and that it could play a physiological role in bone and cartilage *in vivo*.

### Characterization of gross phenotype of the *Elovl6*^*-/-*^ mice

**Fig 1:**
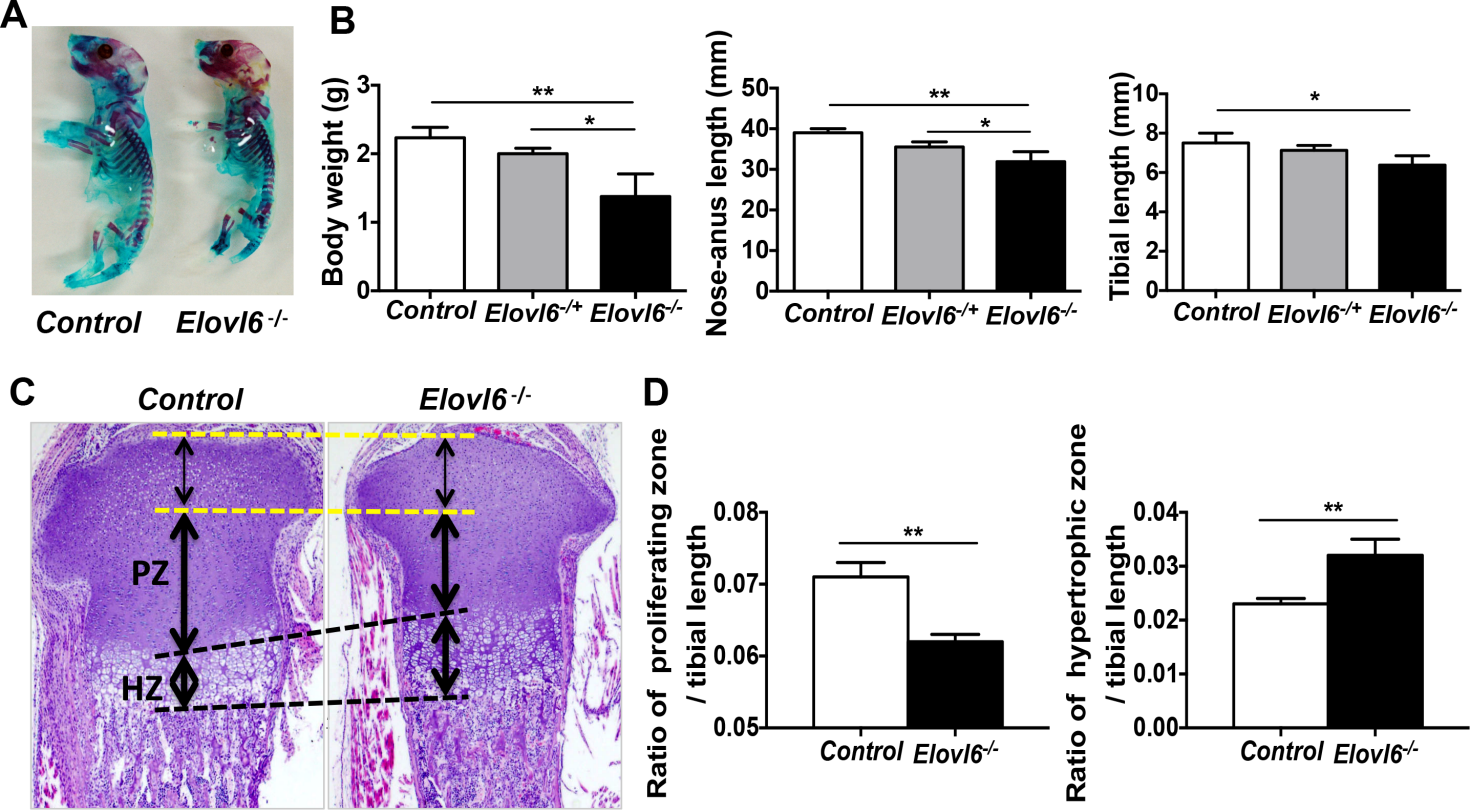
*Elovl6*^*-/-*^ mice were smaller in size and showed abnormality in growth plate. (A) Whole mount skeletal staining was performed using Alizarin red S and Alcian blue stain. (B) *Elovl6*^***-/-***^ mice show significantly reduced body weight (left), nose-anus (middle) and longitudinal tibial (right) length compared with controls (n = 17~18 in each group). (C, D) *Elovl6*^***-/-***^ bones show markedly decreased length of proliferating and increased length of hypertrophic layers. (C) Representative images of proximal tibia of *control* and *Elovl6*^*-/-*^ newborn mice. Hematoxylin and eosin stain; H&E. (D) Quantitative analysis of the length of the proliferating and hypertrophic zones of proximal tibia (n = 15–18 in each group). PZ: proliferating zone, HZ: hypertrophic zone. *p < 0.05, **p < 0.01 vs. controls.

To investigate a role of *Elovl6* in bone and cartilage, whole mount skeletal analysis was first performed in control and *Elovl6*^*-/-*^ littermates at E18.5 and birth. The mutant skeleton was apparently smaller in size than that of control littermates, but did not exhibit any obvious patterning defect and skeletal abnormality (Fig 1A). The mean body weight of *Elovl6*^*-/-*^ newborn mice was significantly reduced compared with that of control and *Elovl6*^*-/+*^ littermates (Fig 1B). In accordance with this reduced body weight of *Elovl6*^*-/-*^ mice, significantly shorter nose-anus and longitudinal tibial length was also observed in *Elovl6*^*-/-*^ newborns than in control and *Elovl6*^*-/+*^ littermates (Fig 1B). These results suggest that deletion of *Elovl6* impairs skeletal development.

### Lack of *Elovl6* exhibited abnormal phenotype in growth plate

Next, histological analysis was performed on proximal tibial bone samples isolated from control and *Elovl6*^*-/-*^ newborn mice. The relative length of the proliferating chondrocyte zone adjusted with the total tibial length was significantly shorter, but that of the hypertrophic zone was longer in *Elovl6*^*-/-*^ newborn mice than in *control* littermates (Fig 1C and 1D). These results show that ablation of *Elovl6* displays abnormal growth plate phenotype in newborn mice, suggesting its potential physiological role in cells of the chondrocyte lineage during embryonic bone development.

### Lack of *Elovl6* impaired proliferation in cells of the chondrocyte lineage *in vivo*

**Fig 2:**
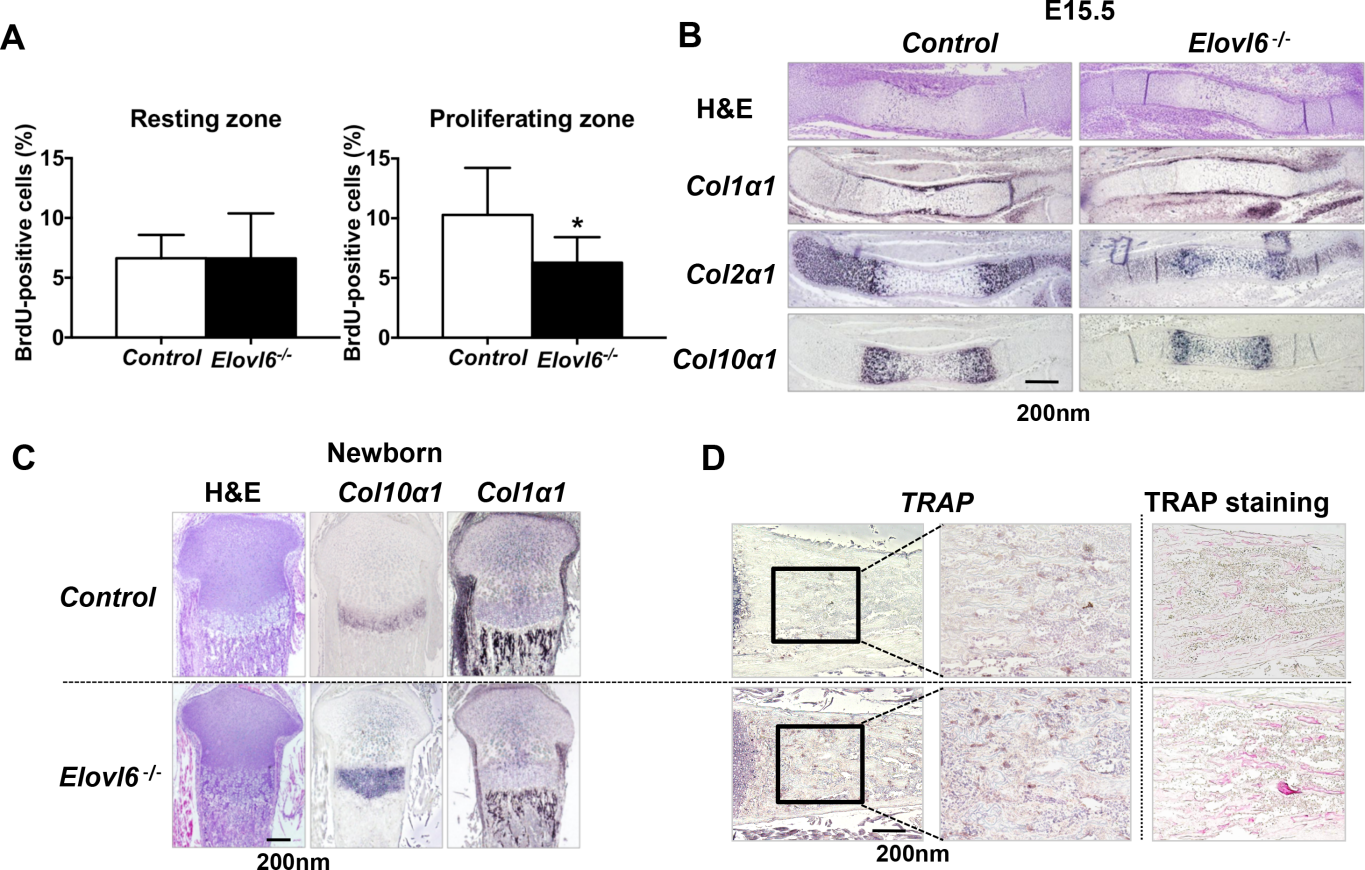
*Elovl6*^*-/-*^ mice exhibited skeletal phenotype *in vivo*. (A) Lack of *Elovl6* leads to the decreased number of BrdU-positive cells in proliferating chondrocyte layer. Quantitative analysis of BrdU-positive cells in the resting and proliferating chondrocyte layers of the proximal tibia (n = 16~18 in each group). (B) Lack of *Elovl6* leads to slight delay in initial chondrocyte differentiation at E15.5. (C) Lack of *Elovl6* leads to expansion of the hypertrophic layer expressing *Col10α1* in growth plate and markedly reduced trabecular bone expressing *Col1α1* at birth. (D) Lack of *Elovl6* leads to the increased number of cells expressing *TRAP* and stained with TRAP in primary spongiosa of proximal tibial samples. *In situ* hybridization analysis was performed on proximal tibial samples using riboprobes of *Col2α1*, *Col10α1*, *Col1α1*, and *TRAP*. *p < 0.05 vs. controls.

The reduced length of the proliferating chondrocyte zone observed in *Elovl6*^*-/-*^ newborn mice could be a consequence of either decreased chondrocyte proliferation, increased apoptosis, or accelerated chondrocyte differentiation. In order to test these possibilities, proliferation of chondrocyte was first examined by BrdU incorporation in E18.5 embryos. In the resting chondrocyte zone of proximal growth plate, the number of BrdU-positive cells was indistinguishable between *control* and *Elovl6*^*-/-*^ embryos; however, in the proliferating chondrocyte zone, the number of BrdU-positive cells was decreased in *Elovl6*^*-/-*^ embryos compared with controls (Fig 2A). No significant difference in the number of apoptotic cells was detected in E18.5 *Elovl6*^*-/-*^ embryos versus (vs.) controls as determined by a terminal deoxynucleotidyl transferase-mediated nick end labeling staining (data not shown). Collectively, these data suggest that the reduced length of proliferating chondrocyte zone could be, at least partially, due to reduced proliferation of cells of the chondrocyte lineage.

### Deletion of *Elovl6* showed acceleration of chondrocyte differentiation and osteoporotic phenotype at birth

To examine a role of *Elovl6* in chondrocyte differentiation *in vivo*, detailed histological and *in situ* hybridization analyses were performed on histological sections of hind limbs isolated from E15.5 and E18.5 embryos and newborn mice. At E15.5, spatially reduced expression of *Col2*α*1* and *Col10*α*1* mRNAs was detected; while, expression of the osteoblastic marker, *Col1*α*1* mRNA, was almost similar to that of controls, and no obvious impaired vessel invasion was observed in *Elovl6*^*-/-*^ embryos compared with controls (Fig 2B). Thus, ablation of *Elovl6* may display a slight, if any, delay in initiation of chondrocyte differentiation.

At birth, expansion of the hypertrophic chondrocyte layer expressing *Col10*α*1* mRNA was observed in growth plate; yet, trabecular bone expressing *Col1*α*1* was significantly reduced in *Elovl6*^*-/-*^ mice compared with control littermates (Fig 2C). Moreover, *Elovl6*^*-/-*^ bones exhibited approximately twice more cells expressing *TRAP* and stained with TRAP than controls in primary spongiosa of proximal tibial samples (Fig 2D). The similar observation was also made at E18.5 (data not shown). There were no significant differences in cartilage polysaccharides including glycogen and proteoglycans detected by PAS and safranin O staining, respectively, between *control* and *Elovl6*^*-/-*^ mice (S2 Fig). Collectively, these results suggest that the reduced length of the proliferating chondrocyte layer and increased length of the hypertrophic zone could be due to reduced proliferation and accelerated differentiation in cells of the chondrocyte lineage. There were no significant changes in polysaccharide and proteoglycan store between *control* and *Elovl6*^*-/-*^ growth plate. Moreover, because the number of osteoclastic cells stained with TRAP was apparently increased in *Elovl6*^*-/-*^ mice, their reduced trabecular bone might be, at least in part, due to increased bone resorption by osteoclasts. Taken together, *Elovl6* may play physiological roles in cells of the chondrocyte, osteoblast, and osteoclast lineages. Hereafter, we focused on examining how Elovl6 modulates chondrocyte biology.

### Deletion of *Elovl6* altered fatty acid composition in primary chondrocytes

**Fig 3:**
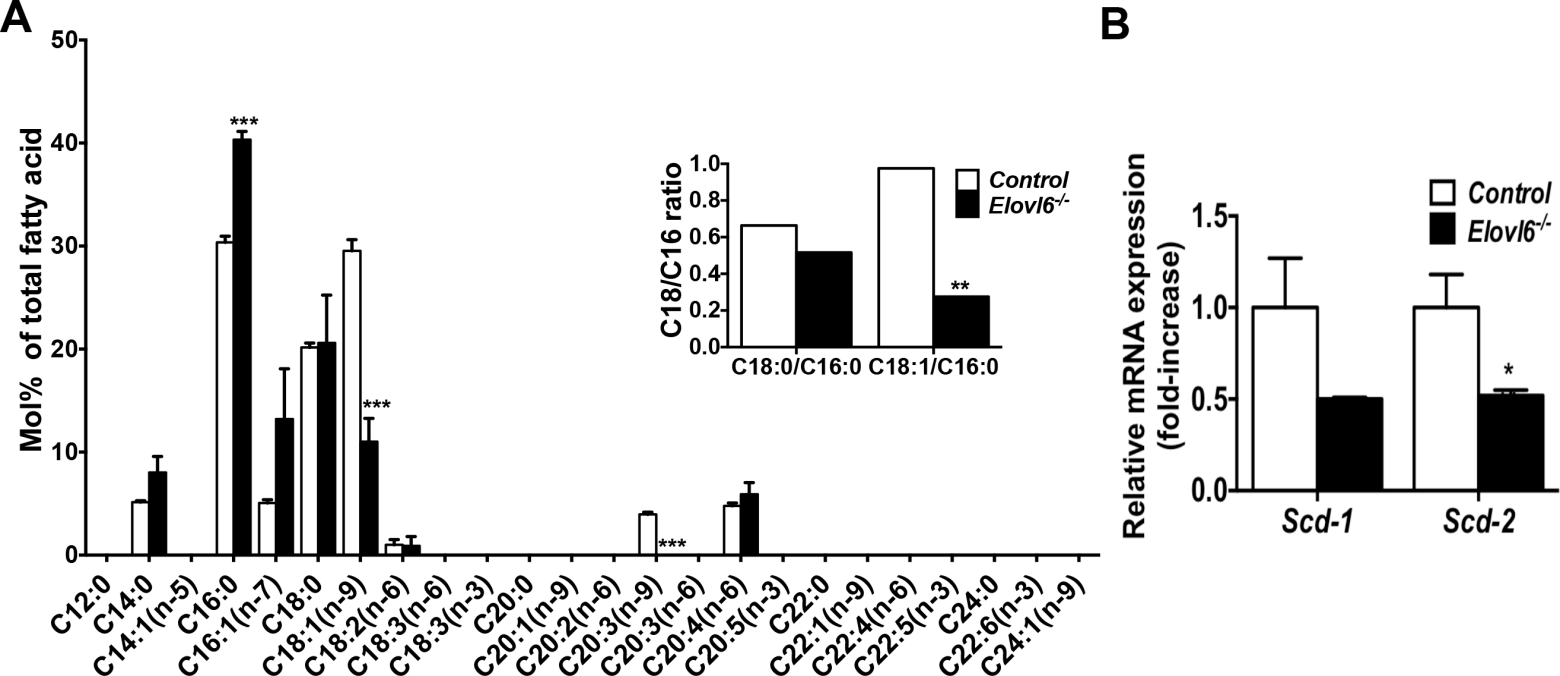
Lack of *Elovl6* altered fatty acid composition in costal primary chondrocytes. (A) Lack of *Elovl6* significantly increases C16:0 and decreases C18:1 (n-9) in primary chondrocytes. Gas chromatography analysis was performed to examine fatty acid composition of primary chondrocytes harvested from *control* and *Elovl6*^*-/-*^ mice (n = 3 in each group). (B) Lack of *Elovl6* significantly decreases *Scd-2* mRNA levels in primary chondrocyte. Quantitative determination of levels of *Scd-1* and *Scd-2* mRNA was examined by real-time qPCR (n = 4 in each group). *p < 0.05, **p < 0.01, ***p < 0.005 vs. controls.

To test whether deletion of *Elovl6* in primary chondrocytes alters their fatty acid composition as we reported previously in liver, serum, and macrophages [5, 6], gas chromatography analysis was performed on total lipid samples isolated from *control* and *Elovl6*^*-/-*^ primary chondrocytes. *Elovl6*^*-/-*^ chondrocytes displayed a significant increase in C16:0 and a decrease in C18:1 n-9 fatty acid composition, resulting in a marked reduction of C18:1/C16:0 ratio compared with controls. There were no significant differences in the C16:1 n-7 and C18:0 fatty acid composition between *control* and *Elovl6*^*-/-*^ mice (Fig 3A). Moreover, the ratio of C18:0/C18:1, but not C16:0/C16:1, was significantly higher in *Elovl6*^*-/-*^ chondrocytes than *control*s (the ratio of C18:0/C18:1; control, 0.69 ± 0.04 (n = 3) vs. *Elovl6*^*-/-*^, 1.93 ± 0.36 (n = 3), *p<0.05); this suggests reduced fatty acid desaturation in *Elovl6*^*-/-*^ vs. *control* chondrocytes. Expression of *stearoyl-CoA desaturace* (s*cd)-2* was also significantly decreased in *Elovl6*^*-/-*^ chondrocytes compared with *controls* (Fig 3B). These results, thus, suggest that *Elovl6* plays a role in regulating fatty acid composition and could also modify fatty acid desaturation by modulating *scd-2* mRNA levels and its function in cells of the chondrocyte lineage.

### Lack of *Elovl6* increases expression of chondrocyte differentiation marker *Col10a1* and its related transcriptional regulators, *Foxa2* and *Mef2c*, in primary chondrocytes

**Fig 4:**
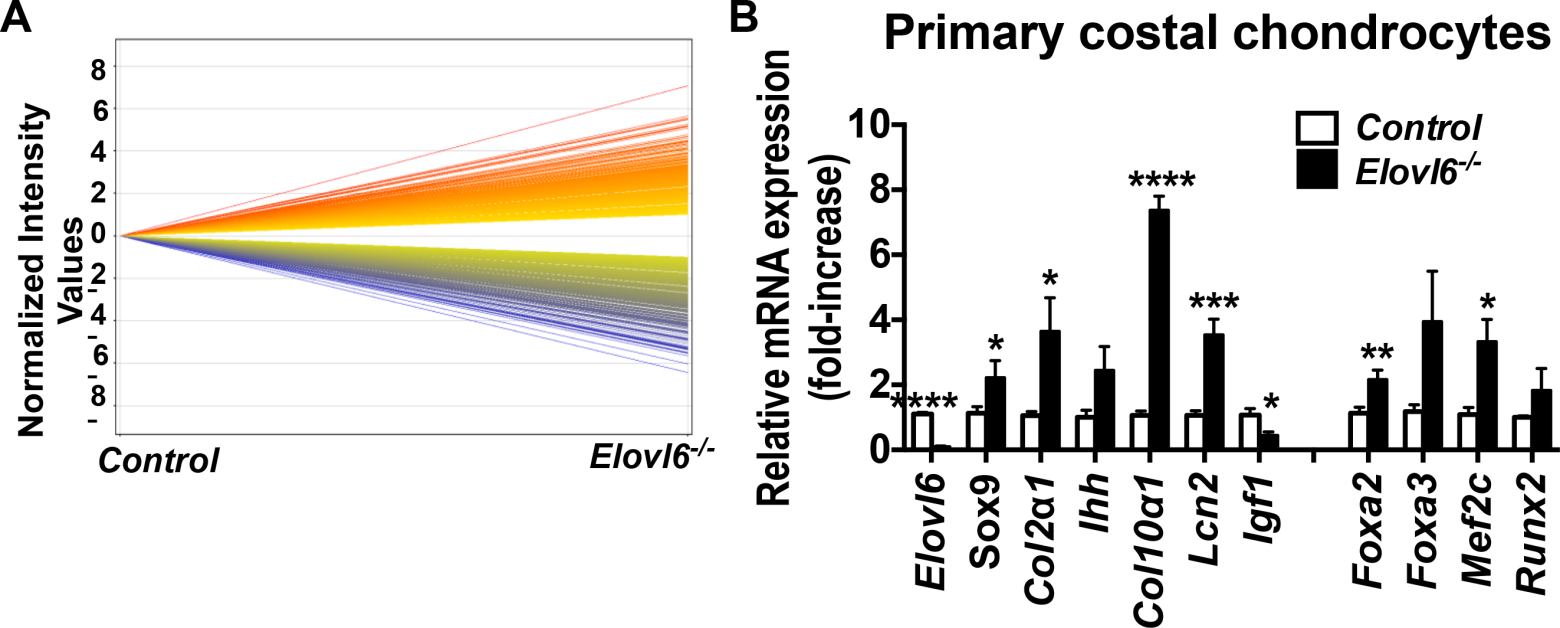
Lack of *Elovl6* increased expression of various chondrocyte differentiation markers and related transcriptional regulators in primary chondrocytes. (A) The summary of microarray analysis of primary chondrocyte. The genes up-regulated in *Elovl6*^*-/-*^ chondrocytes vs. controls were indicated in red; while those down-regulated in *Elovl6*^*-/-*^ vs. controls cells were shown in blue. *Elovl6*-deficient primary chondrocytes showed 1646 up-regulated genes and 2562 down-regulated ones by more than twofold compared with controls. (B) Relative mRNA levels of marker genes for chondrocyte differentiation, were determined by real-time qPCR (n = 3–9 in each group). *p < 0.05, **p < 0.01, ***p < 0.005, ****p<0.001 vs. controls.

To elucidate a molecular mechanism by which *Elovl6*^*-/-*^ mice exhibited abnormal growth plate phenotype, microarray analysis was performed using primary chondrocytes isolated from control and *Elovl6*^*-/-*^ newborn mice (Fig 4A). Table 1 showed a list of genes, which are known to govern chondrocyte physiology and were either up- or down-regulated by more than 2^5^-fold in *Elovl6*^*-/-*^ vs. control primary chondrocytes. The highest levels of mRNA expression were observed in *Col10*α*1* followed by *Scl2a5* (fructose transporter) and *Lcn2*; while, the lowest levels were detected in *Elovl6* followed by *Wnt10a*, *Dkk3*, *and Igf1* in *Elovl6*^*-/-*^ vs. control primary chondrocytes (Table 1 and S1 Table).

**Table 1:** Summary of Microarray analysis.

KO vs. Control	Description	Gene Symbol	Log2 Ratio	Control: gScale Signal	Elovl6 KO: gScale Signal	Primary Accession
Up-regulation	Mus musculus collagen, type X, alpha 1, mRNA	Col10α1	6.89	930.2	110,157.9	NM_009925
	Mus musculus solute carrier family 2 (facilitated glucose transporter), member 5, mRNA	Slc2α5	5.46	54.0	2,327.9	NM_019741
	Mus musculus lipocalin 2, mRNA	Lcn2	5.02	459.3	14,930.1	NM_008491
Down-regulation	Mus musculus insulin-like growth factor 1, transcript variant 1, mRNA	Igf1	-5.08	4,431.7	1,133.0	NM_010446
	Mus musculus dickkopf homolog 3 (Xenopus laevis), mRNA	Dkk3	-5.46	41,224.0	939.2	NM_015814
	Mus musculus wingless related MMTV integration site 10a, mRNA	Wnt10a	-5.53	14,751.5	320.3	NM_009518
	Mus musculus ELOVL family member 6, elongation of long chain fatty acids, mRNA	Elovl6	-6.61	3,657.9	37.4	NM_130450

Genes are listed, which are known to govern chondrocyte physiology and were either up- or down-regulated by more than 2^5^-fold in *Elovl6*^*-/-*^ vs. control primary chondrocytes.

To test these results of microarray analysis and examine expression of various markers for chondrocyte differentiation, real-time qPCR analysis was next performed. The relative levels of expression of *Sox9*, *Col2*α*1*, *Col10*α*1*, and *Lcn2* mRNA were significantly increased in *Elovl6*^*-/-*^ cells compared with those in control cells (relative *Sox9* mRNA expression, *Elovl6*^*-/-*^ cells, 2.2±0.5 vs. control cells, 1.1±0.2. *Col2*α*1* mRNA expression for *Elovl6*^*-/-*^ cells, 3.6±1.0 vs. control cells, 1.1±0.1. *Col10*α*1* mRNA expression for *Elovl6*^*-/-*^ cells, 7.4±1.0 vs. control cells, 1.1±0.1. *Lcn2* mRNA expression for *Elovl6*^*-/-*^ cells, 3.5 ± 0.5 vs. control cells, 1.1 ± 0.1). While, the relative levels of *Igf1* expression were significantly reduced to 45% in *Elovl6*^*-/-*^ cells compared with those in control cells (Fig 4B). Moreover, the expression of *Foxa2* and *Mef2c*, positive transcriptional regulators for *Col10α1*, was also significantly increased in *Elovl6*^*-/-*^ primary chondrocytes compared with controls (relative levels of *Foxa2* mRNA expression for *Elovl6*^*-/-*^ cells, 2.2±0.3 vs. control cells, 1.1±0.2. *Mef2c* mRNA expression for *Elovl6*^*-/-*^ cells, 3.3±0.7 vs. control cells, 1.1±0.2). There was no significant difference in cell number when 1.0x10^4^ cells/cm^2^ of *control* and *Elovl6*^*-/-*^ primary chondrocytes were seeded on day 0 and incubated for 7 days in a growth medium (*control*, 5.5±0.7 (n = 6) vs. *Elovl6*^*-/-*^, 4.7±0.3 x10^4^ cells/cm^2^ (n- = 8)). Thus, these data suggest that ablation of *Elovl6* might primarily accelerate chondrocyte differentiation rather than directly reduce proliferation in cells of the chondrocyte lineage. Furthermore, consistent with elongated hypertrophic chondrocyte layers expressing *Col10*α*1*, the highest levels of relative expression of *Col10*α*1* mRNA was detected in both microarray and real-time qPCR analyses among other chondrocyte differentiation markers in *Elovl6*^*-/-*^ chondrocytes compared with controls; this increased levels of *Col10*α*1* expression could be presumably associated with elevated levels of *Foxa2* and *Mef2c* expression.

### Knockdown of *Elovl6* altered fatty acid composition in ATDC5 cells

**Fig 5:**
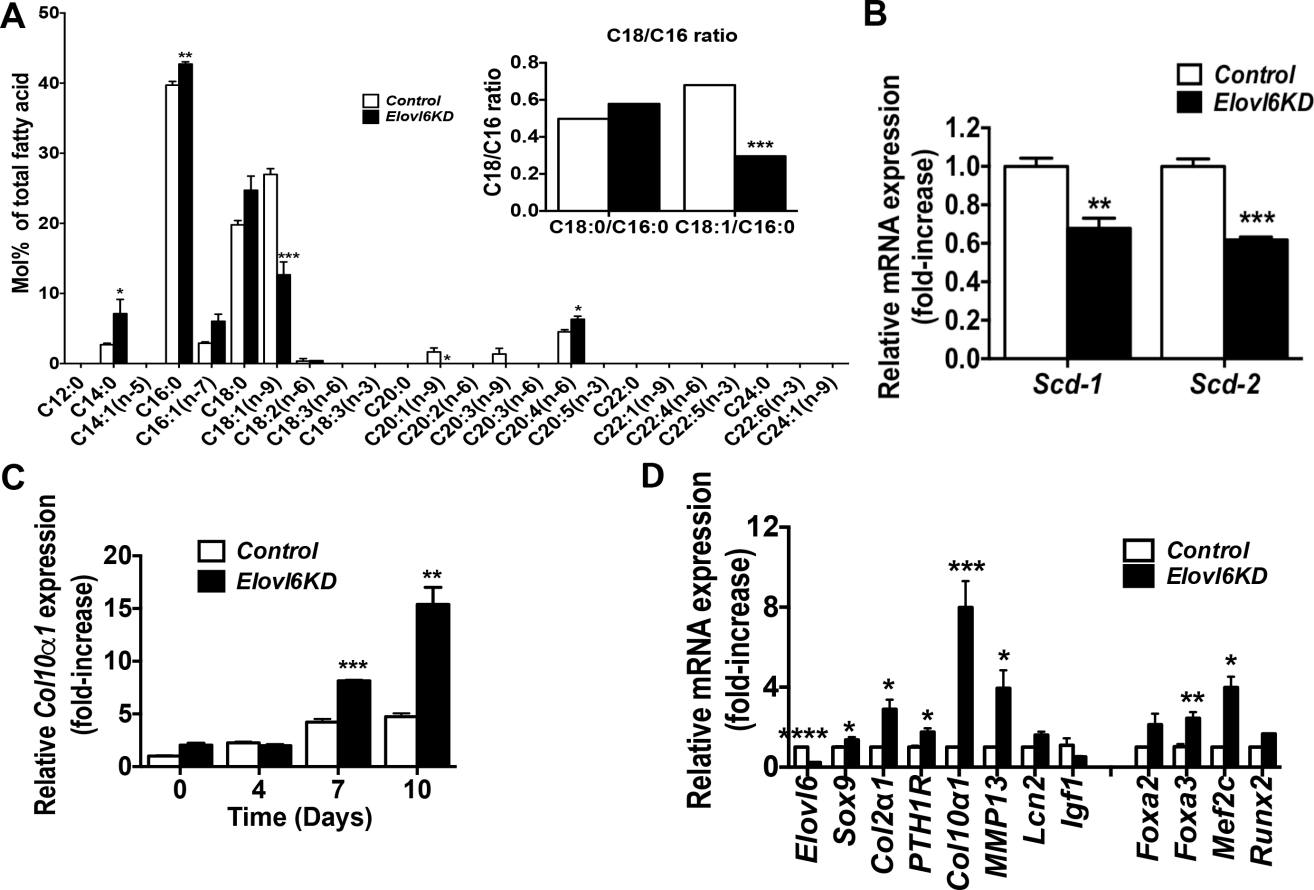
Knockdown of *Elovl6* in mouse chondrogenic ATDC5 cells modified levels of expression of *Col10α1* and its related transcription factors. (A) Knockdown of *Elovl6* significantly increases C16:0 and decreases C18:1 (n-9) in ATDC5 cells (n = 4 in each group). (B) Knockdown of *Elovl6* significantly decreases expression of *Scd-1* and *Scd-2* in ATDC5 cells (n = 3 in each group). (C) Knockdown of *Elovl6* significantly increases gene expression of *Col10α1* in a time-dependent manner. Control and *Elovl6*-KD ATDC5 cells were cultured in a differentiation medium for 0, 4, 7, and 10 days after cells reached a plateau. (D) Relative mRNA levels of marker genes for chondrocyte differentiation, were determined by real-time qPCR (n = 3–9 in each group). *p < 0.05, **p < 0.01, ***p < 0.005, ****p<0.001 vs. controls.

To further examine mechanistic insights underlying increased expression of *Col10α1* and its associated genes, *Foxa2* and *Mef2c*, in *Elovl6*^*-/-*^ chondrocytes, four clonal mouse chondrogenic ATDC5 cell lines stably expressing *Elovl6* shRNA and three independent ATDC5 cell lines expressing *control* shRNA were established. Expression of *Elovl6* assessed by real-time qPCR using two independent sets of primers was reduced to approximately 20% in two independent *Elovl6* knockdown (KD) ATDC5 cell lines (KD-5 and KD6), and about 40 and 50% in *Elovl6*-KD cell lines, KD-2 and KD-7, respectively, compared with control cells (S3 Fig). Hereafter we used the former two *Elovl6*-KD cell lines in our experiments. The analysis of fatty acid composition showed an increase of C16:0 and a decrease of C18:1 n-9, resulting in a significantly reduced C18:1/C16:0 ratio in *Elovl6*-KD vs. control cells (Fig 5A). Moreover, the ratio of C18:0/C18:1, but not C16:0/C16:1, was significantly higher in *Elovl6*-KD chondrocytes than *control*s (the ratio of C18:0/C18:1; control, 0.74 ± 0.04 (n = 3) vs. *Elovl6*-KD, 1.96 ± 0.20 (n = 3), ***p<0.005); this suggests reduced fatty acid desaturation in *Elovl6*-KD vs. *control* chondrocytes. Expression of both *Scd-1* and *Scd-2* was also lower in *Elovl6-KD* cells than controls (Fig 5B). These results were consistent with those shown above in primary *Elovl6*^*-/-*^ chondrocytes vs. controls, and, therefore, confirmed the sufficient ablation of the *Elovl6* gene in the *Elovl6*-KD ATDC5 cells.

### Knockdown of *Elovl6* promoted nuclear translocation of Foxa2 in cells of the chondrocyte lineage

There was again no significant difference in cell number when 1.0x10^4^ cells/cm^2^ of *control* and *Elovl6-*KD ATDC5 cells were seeded on day 0 and incubated in a growth medium for 1, 3, and 7 days (on day 7; *control*, 13.3±0.7 vs. *Elovl6-KD*, 14.7±0.8 x10^4^ cells/cm^2^). An effect of *Elovl6* on *Col10α* expression was next tested in ATDC5 cells by incubating cells in a differentiation medium for 0, 4, 7, and 10 days after cells reached a plateau. Knockdown of *Elovl6* showed a significant increase in *Col10*α*1* mRNA expression in a time-dependent manner (Fig 5C). Moreover, *Elovl6*-KD cells displayed a significant increase in mRNA expression of Sox9, *Col2*α*1*, *PTH1R*, *Col10*α*1*, *MMP13*, *Foxa3*, and *Mef2c* when cells were induced chondrocyte differentiation by incubating cells in a differentiation medium for 7 days (Fig 5D). Those results were consistent with our observation above in *control* and *Elovl6*^*-/-*^ primary chondrocytes.

**Fig 6:**
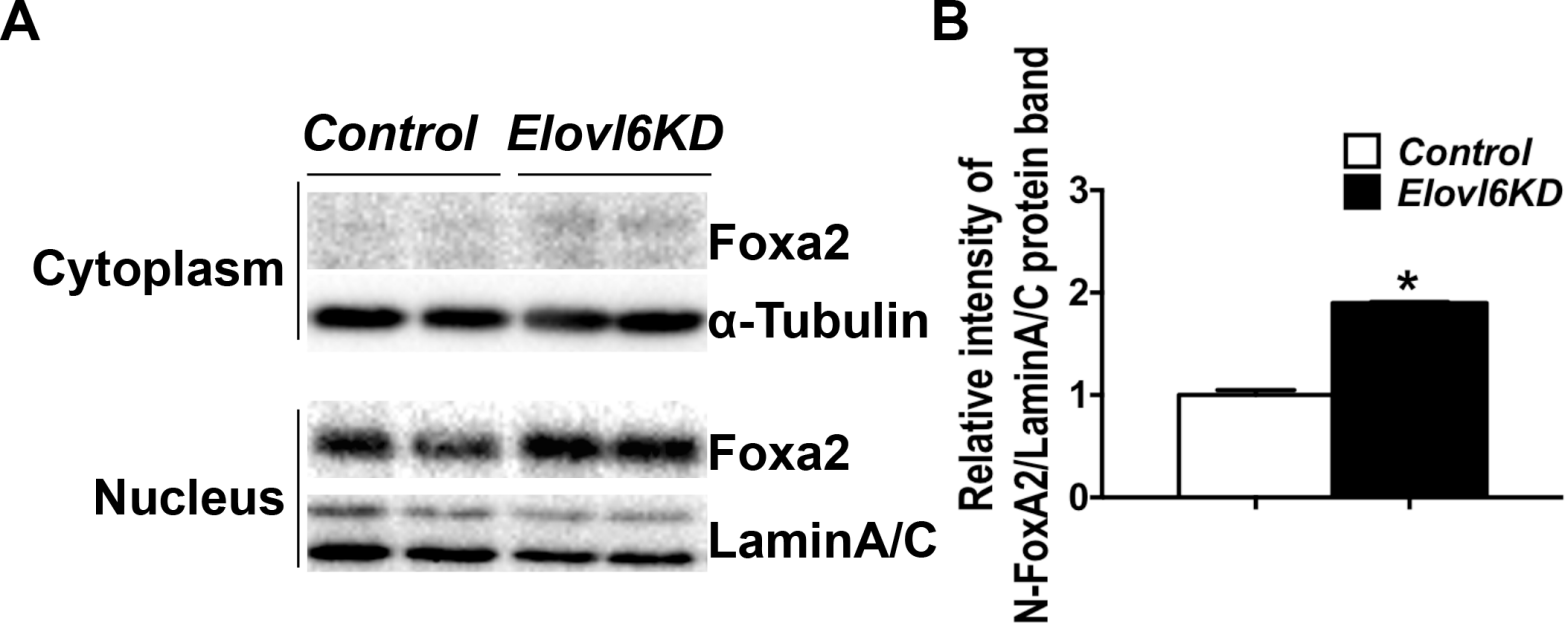
Knockdown of *Elovl6* in ATDC5 cells increased nuclear localization of Foxa2. (A) Western blot analysis of Foxa2 using cytoplasmic and nuclear extracts isolated from control and *Elovl6*-KD ATDC5 cells after an incubation of the cells in a differentiation medium for 7 days. (B) Quantitative analysis of the intensity of Foxa2 bands in reference to those of Lamin A/C. *p < 0.05 vs. controls.

Foxa2 is a critical transcription factor, which has been shown to regulate *Col10*α*1* mRNA levels by directly binding to a conservative sequence of its enhancer lesion in nucleus. Therefore, protein levels of Foxa2 in the cytoplasm and nucleus of *Elovl6*-KD and *control* cells were next examined by Western blot analysis (Fig 6A). The relative intensity of the Foxa2 protein band in the nuclear fraction was significantly increased in *Elovl6*-KD vs. *control* cells (Fig 6B); while, those in the cytoplasm fraction were almost indistinguishable between the two groups (Fig 6A). Collectively, these data suggest that knockdown of *Elovl6* accelerated chondrocyte differentiation and that increased *Col10*α*1* mRNA levels were likely through a mechanism, at least partially, mediated by increased *Foxa3* and *Mef2c* expression and nuclear Foxa2 localization in cells of the chondrocyte lineage.

### Knockdown of *Elovl6* increased cytoplasmic localization of HDAC4/5/7 in cells of the chondrocyte lineage

**Fig 7:**
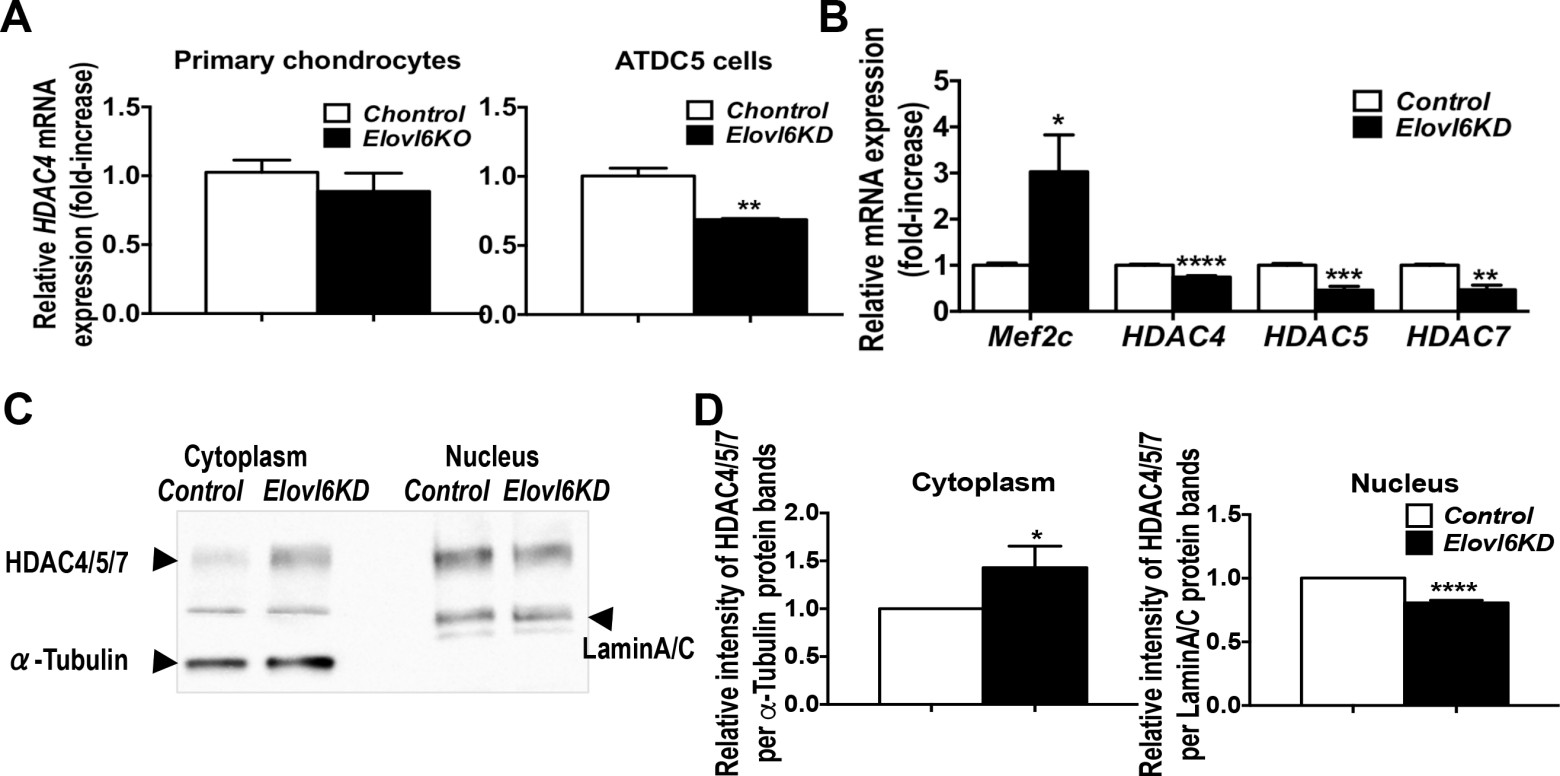
Knockdown of *Elovl6* in ATDC5 cells promoted cytoplasmic localization of HDAC4/5/7. (A, B) Knockdown of *Elovl6* significantly decreases expression of *HDACs 4* (A, B), *5* and *7* (B) mRNA in ATDC5 cells (n = 3 in each group). (C) Representative images of Western blot analysis of HDAC4/5/7 using cytoplasmic and nuclear extracts isolated from control and *Elovl6*-KD ATDC5 cells after an incubation of the cells in a differentiation medium for 7 days. (B) Quantitative analysis of the intensity of HDAC4/5/7 bands in reference to those of α-Tubulin for cytoplasmic and Lamin A/C for nuclear extracts. *p < 0.05, **p < 0.01, ***p < 0.005, ****p<0.001 vs. controls.

It has been shown that *Mef2c* expression is regulated by protein localization of class II HDACs [19–21]. For example, when HDAC4 phospho-serine 246 is dephosphorylated by protein phosphatase 2A (PP2A), this enhances nuclear translocation of HDAC4 and thereby inhibits *Mef2c* transcription [22]. Thus, we next tested whether knockdown of *Elovl6* alters mRNA expression of *HDACs 4*, *5*, and *7* and their cellular localization in cells of the chondrocyte lineage. *Elovl6*^*-/-*^ primary chondrocytes showed about 75% reduction in *HDAC7* expression compared with controls cells in microarray analysis (Log2 Ratio, -1.72 vs. controls) although they did not display significant changes in *HDAC4* in the array and real-time qPCR analyses (Fig 7A and S1 Table). Knockdown of *Elovl6* showed significantly reduced *HDACs 4*, *5*, and *7* mRNA levels when *Mef2c* expression was markedly elevated in ATDC5 cells (Fig 7A and 7B). Moreover, the relative intensity of the HDAC4/5/7 protein bands in the cytoplasmic fraction was increased significantly; whereas, those in the nuclear fraction were reduced in *Elovl6*-KD vs. *control* cells (Fig 7C and 7D). These results suggest that knockdown of *Elovl6* might regulate *Mef2c* expression by modulating cellular localization of class II HDACs and thus chondrocyte hypertrophy.

### Palmitic and stearic acids in a medium modified levels of *Col10α1*, *Foxa3*, and *Mef2c* mRNA expression in *Elovl6*-KD ATDC5 cells

**Fig 8:**
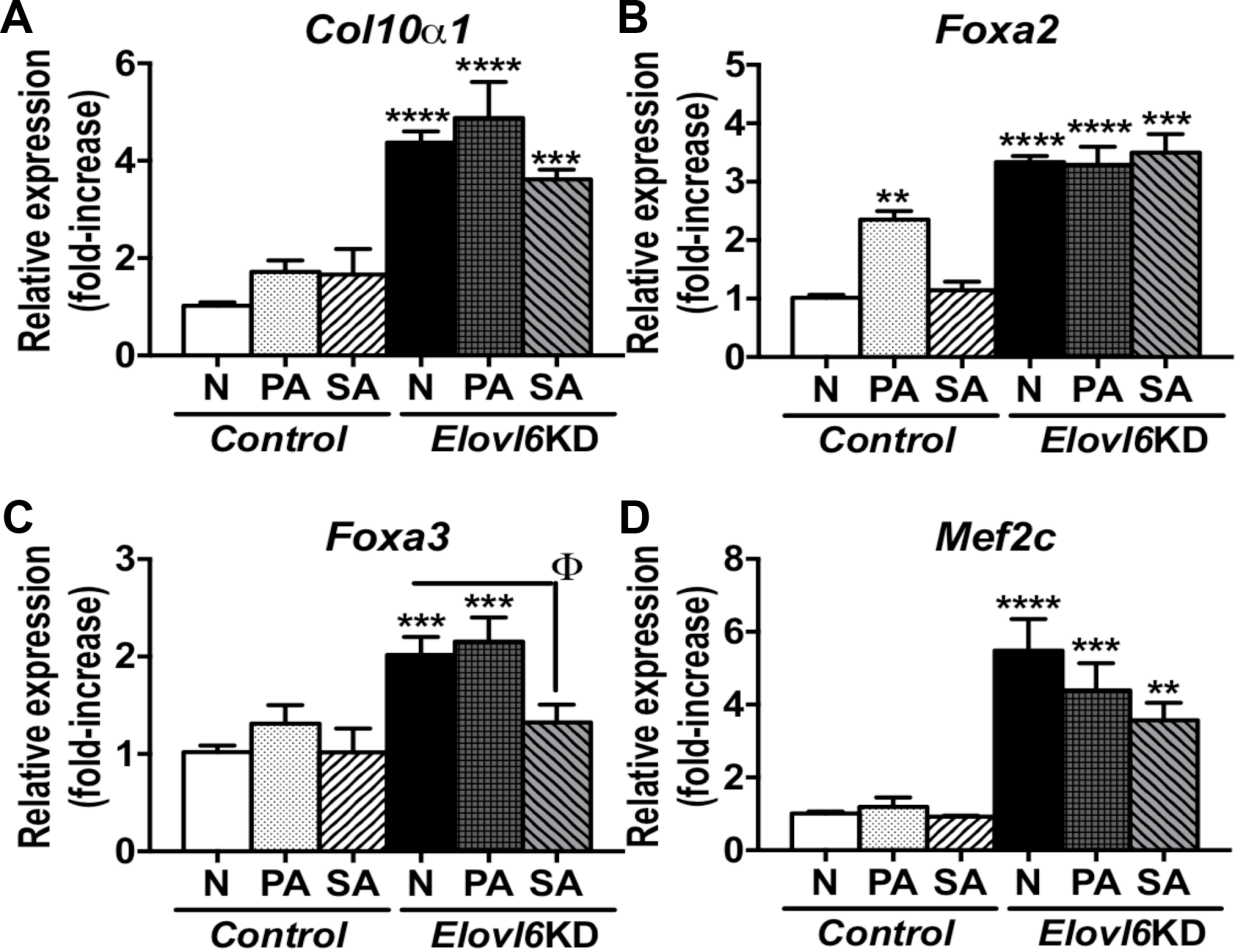
Palmitic or stearic acid in a medium could affect levels of *Col10α1*, *Foxa3*, and *Mef2c* mRNA expression in *Elovl6*-KD ATDC5 cells. *Control* and *Elovl6*-KD ATDC5 cells were incubated in a modified differentiation medium supplemented with either 0μM fatty acid (N), 50μM palmitic acid (PA), or 50μM stearic acid (SA) for 7 days. Expression of (A) *Col10α1*, (B) *Foxa2*, (C) *Foxa3*, and (D) *Mef2c* mRNA was examined by real-time qPCR. *p < 0.05, **p < 0.01, ***p < 0.005, ****p<0.001 vs. controls. ^ϕ^p < 0.05 vs. *Elovl6*-KD cells.

To examine how fatty acids in a medium affect expression of markers for chondrocyte differentiation, *control* and *Elovl6*-KD ATDC5 cells were incubated in a modified differentiation medium containing either 0μM fatty acid, 50 μM palmitic acid, or 50 μM stearic acid for 7 days when *Col10a1* mRNA levels were significantly increased in *Elovl6*-KD cells vs. controls (Fig 5C). Palmitic acid significantly increased *Foxa2* mRNA in control cells; yet, it did not alter elevated *Col10α1*, *Foxa2*, *Foxa3*, and *Mef2c* expression in *Elovl6*-KD cells (Fig 8). Stearic acid markedly reduced increased mRNA levels of *Foxa3*, and slightly did those of *Col10α1* and *Mef2c* in *Elovl6*-KD cells (Fig 8A, 8C and 8D). These results suggest that the fatty acid composition such as the C16:0/C18:0 ratio in a medium could also modify levels of *Col10α1*, *Foxa3*, and *Mef2c* mRNA expression in *Elovl6*-KD ATDC5 cells.

## Discussion

Here we report the novel finding that *Elovl6* is important in embryonic endochondral bone development *in vivo*. The study began with our initial observation that the *Elovl6*^*-/-*^ mice often suffered from growth retardation despite their significant metabolic advantages in insulin sensitivity and atherosclerosis [5, 6]. Ablation of *Elovl6* exhibited reduced proliferating and elongated hypertrophic chondrocyte layers in growth plate and reduced trabecular bone with the increased number of osteoclast-like cells in the primary spongiosa of the proximal tibia. This bone phenotype was suggestive of an importance of *Elovl6* in cells of the chondrocyte, osteoblast, and osteoclast lineages.

Direct effect of *Elovl6* on chondrocyte proliferation was inconclusive in the present study. *Elovl6*^*-/-*^ growth plates showed reduced length of proliferating chondrocyte layer. The number of BrdU-positive cells was significantly decreased in *Elovl6*^*-/-*^ vs. control embryos in the proliferating chondrocyte layer of proximal growth plate; however, there was no marked difference in cell number of *Elovl6*^*-/-*^ and *Elovl6*-KD vs. control chondrocytes *in vitro*. Notably, the sizes and structures of endochondral bones depend on the coordinated regulation of chondrocyte proliferation, maturation, and hypertrophy in response to multiple extracellular signals including indian hedgehog (Ihh) and parathyroid hormone related peptide (PTHrP) [23]. In brief, PTHrP acts on chondrocytes to keep the chondrocytes proliferating and delay their differentiation into prehypertrophic and hypertrophic chondrocytes. When chondrocytes stop proliferation, they synthesize Ihh, which, in turn, acts to increase the synthesis of PTHrP. Therefore, our *in vivo* observation could be an overall consequence of the complex non-autonomous regulation of chondrocyte proliferation by Elovl6 and other signals; this could not be reproduced in the autonomous *in vitro* system. Interestingly, the active form of hedgehog protein is shown to be lipid modified with a palmitate group at its amino terminus and a cholesterol moiety at its carboxyl terminus [24]. The biological function of palmitoylation of hedgehog protein has not been fully understood; yet, palmitoylated sonic hedgehog was, for instance, reported to increase its potency in 10T1/2 mouse fibroblasts [25]. In our microarray analysis, we observed approximately 80% reduction of mRNA levels of *hedgehog acyltransferase (HHAT)*, which governs palmitoylation of hedgehog (Log2 Ratio, -2.51 vs. controls); whereas, there was about a twofold increase in *Gli1*, a downstream positive target of hedgehog signaling (Log2 Ratio, 1.01 vs. controls), in *Elovl6*-null primary chondrocytes vs. controls. Because lack of *Elovl6* increases relative C16:0 fatty acid composition in chondrocytes, it might be possible to modify cholesterol moiety and promote palmitoylation of Ihh and modify its biological function in chondrocyte proliferation and hypertrophic conversion in *Elovl6*^*-/-*^ growth plate vs. controls. In this regard, however, further studies will be required to test this possibility.

At E15.5 when vessel and perichondrium osteoblast invasion starts [23], we observed a slight delay in chondrocyte differentiation in *Elovl6*^*-/-*^ embryos compared with controls. Microarray analysis showed a marked decrease in *Wnt10a* mRNA levels in *Elovl6*^*-/-*^ primary chondrocytes. Wnt10a is suggested to regulate the apical ectodermal ridge formation, which is essential for limb morphogenesis [26]. Given the lower birth rate for *Elovl6*^*-/-*^ than control mice, possibly impaired embryonic limb development earlier than E15.5 in *Elovl6*^*-/-*^ embryos could result in an apparent delay in initiation of chondrocyte differentiation of surviving *Elovl6*^*-/-*^ embryos. Despite this observation at E15.5, the most striking phenotype in *Elovl6*^*-/-*^ growth plate was an expansion of hypertrophic chondrocyte zones expressing *Col10α1* at birth. Cell-based *in vitro* assay also supported increased chondrocyte differentiation in *Elovl6*^*-/-*^ and *Elovl6*-KD cells. The potential underlying mechanisms, by which *Col10α1* expression increased significantly, were elevated expression of *Foxa2* and/or *Foxa3* and *Mef2c*, and increased nuclear localization of Foxa2. Transcription factors, Foxa2/3 and Mef2c, are known to directly modify *Col10α1* expression and promote chondrocyte hypertrophy *in vivo* [27–29]. It was shown previously that Foxa2 is a critical transcription factor, which regulates both lipid and glucose metabolism by modulating expression of various genes including *scd-1*, *fatty acid synthase*, and *glucose 6-phosphatase* in liver [27]. Our data also demonstrated that *Elovl6*-mediated changes in fatty acid composition modify expression and cellular localization of Foxa2. Therefore, Foxa2, not limited to, could be, at least in part, a critical regulator for lipid metabolism in *Elovl6*-null cells of the chondrocyte lineage. Moreover, a line of evidence has shown that phosphorylation of HDAC4/5 at three conserved serines promotes their association with 14-3-3 proteins in the cytoplasm [21]; PTHrP promotes PP2A-mediated dephosphorylation of HDAC4 and its translocation to nucleus, and thereby inhibits Mef2 transcription and chondrocyte hypertrophy [22]. Our data demonstrated that knockdown of *Elovl6* could increase *Mef2c* expression, at least in part, by inhibiting nuclear translocation of class II HDACs in cells of the chondrocyte lineage. Collectively, it would be critical to next address how the change of the C18/C16 fatty acid ratio promotes expression and nuclear translocation of Foxa2, and cytoplasmic localization of class II HDACs in future studies. The potential mechanisms could include palmitoylation, as we mentioned above regarding Ihh, some unknown lipid mediator, or altered signaling presumably through phospholipids of cellular membranes. Furthermore, incubation of *Elovl6*-KD cells in a medium supplemented with either palmitic or stearic acid modified expression of *Col10α1*, *Foxa3*, and *Mef2c* mRNA. Although cartilage is typical avascular tissue, our data suggest a possibility that fatty acid composition in intracellular or extracellular fluid could, at least partially, modify expression of various genes related to chondrocyte biology and an overall growth plate phenotype particularly after vascular invasion.

We found a significant reduction of levels of *Igf1* mRNA in *Elovl6*^*-/-*^ primary chondrocytes compared with controls. Ablation of *Igf1* in cells of the chondrocyte lineage, however, previously showed lack of late terminal hypertrophic chondrocyte layer and reduced glycogen store [30]. Because this phenotype was not observed in *Elovl6*^*-/-*^ growth plate, we anticipate that this significantly reduced *Igf1* mRNA levels in *Elovl6*^*-/-*^ primary chondrocytes would be the secondary response to *Elovl6*-mediated altered fatty acid composition in chondrocytes.

Lastly, the present study addressed mainly a role of Elovl6 in cells of the chondrocyte lineage; however, *Elovl6*^*-/-*^ mice exhibited osteoporotic bone phenotype and increased osteoclast-like cells in primary spongiosa of proximal tibia. Thus, we anticipate that *Elovl6* in cells of the osteoblast and osteoclast lineages would also play a crucial role in bone formation and remodeling. In summary, this study demonstrated that *Elovl6*-mediated fatty acid composition would be critical for embryonic growth plate development.

## Supporting Information

S1 Fig *Elovl6* is expressed in cells of the chondrocyte, osteoblast, and osteoclast lineages. (A) To test expression of *Elovl6* in bone, *in situ* hybridization analysis was performed on histological sections of proximal tibias isolated from newborn control mice using sense and anti-sense riboprobes of mouse *Elovl6*. (B) Levels of *Elovl6* mRNA expression were tested by real-time qPCR in the liver, brain, bone, and cells of chondrocyte, osteoblasts, and osteoclast lineages. *p<0.05 vs liver.
(PDF)

S2 Fig No significant differences were shown in cartilage polysaccharides and proteoglycans between *control* and *Elovl6*^-/-^ mice. Cartilage polysaccharides and proteoglycans were detected by (A) PAS and (B) safranin O staining, respectively, between *control* and *Elovl6*^*-/-*^ mice.
(PDF)

S3 Fig Basal characterization of marker gene expression for chondrocyte differentiation among four established *Elovl6*-KD vs. three control cell lines. Four clonal ATDC5 cell lines (KD-2, 5, 6, and 7) were established using *shElovl6*. Cells were incubated in a differentiation medium for 7 days and expression of *Col10α1*, *Foxa2*, *Foxa3*, and *Mef2c* were assessed by real-time qPCR. Based on this result, we used two cell lines, KD-5 and KD-6, whose *Elovl6* expression were reduced to about 20% of that of control cells, for our experiments.
(PDF)

S1 Table Microarray analysis of *control* and *Elovl6*^*-/-*^ primary chondrocytes. All the genes are listed, which were either up- or down-regulated by more than 2^1.5^-fold in *Elovl6*^*-/-*^ vs. control primary chondrocytes.
(XLSX)
