# Supplementary figures and images for: Crucial Role of Elovl6 in Chondrocyte Growth and Differentiation during Growth Plate Development in Mice
Source: PLoS One. 2016 Jul 28;11(7):e0159375. doi: 10.1371/journal.pone.0159375 (PMC4965081; doi:10.1371/journal.pone.0159375)

## S1 Figure

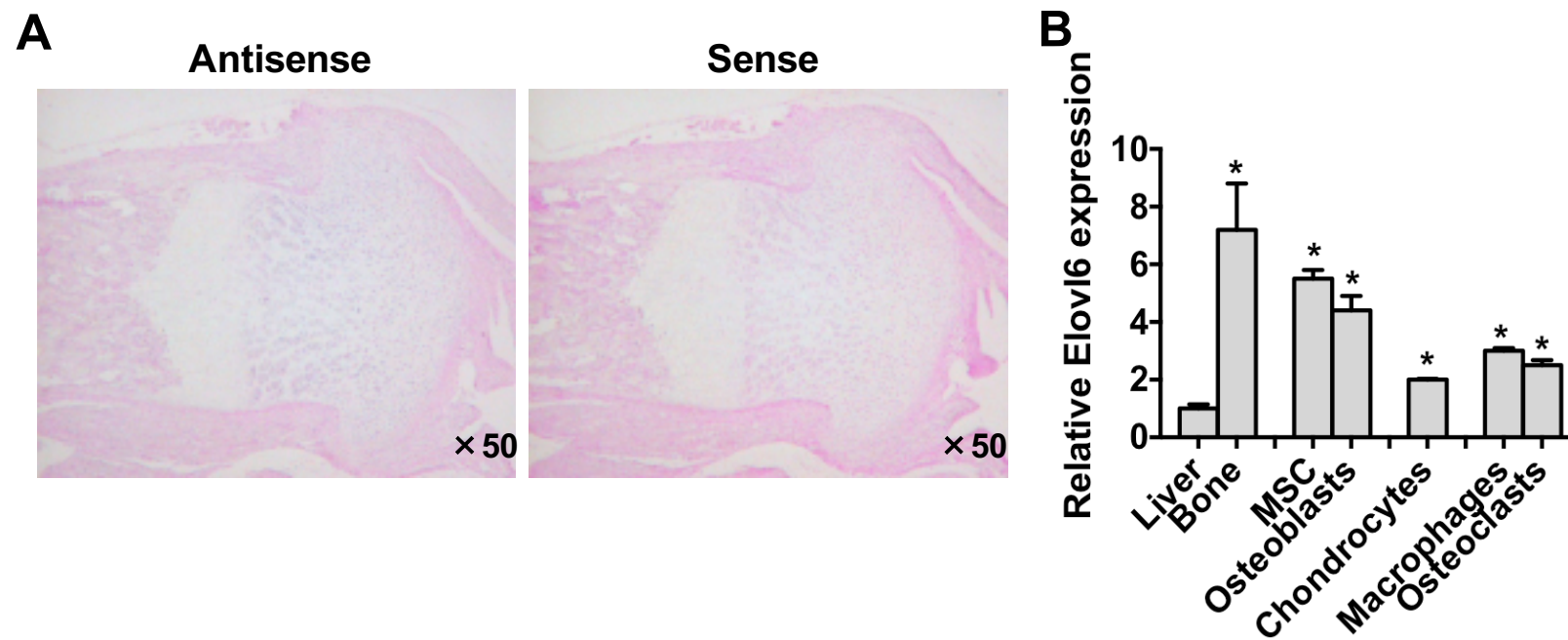

Supplement: S1 Fig — (A) To test expression of Elovl6 in bone, in situ hybridization analysis was performed on histological sections of proximal tibias isolated from newborn control mice using sense and anti-sense riboprobes of mouse Elovl6. (B) Levels of Elovl6 mRNA expression were tested by real-time qPCR in the liver, brain, bone, and cells of chondrocyte, osteoblasts, and osteoclast lineages. *p<0.05 vs liver. (PDF) [file pone.0159375.s001.pdf]

## S2 Figure

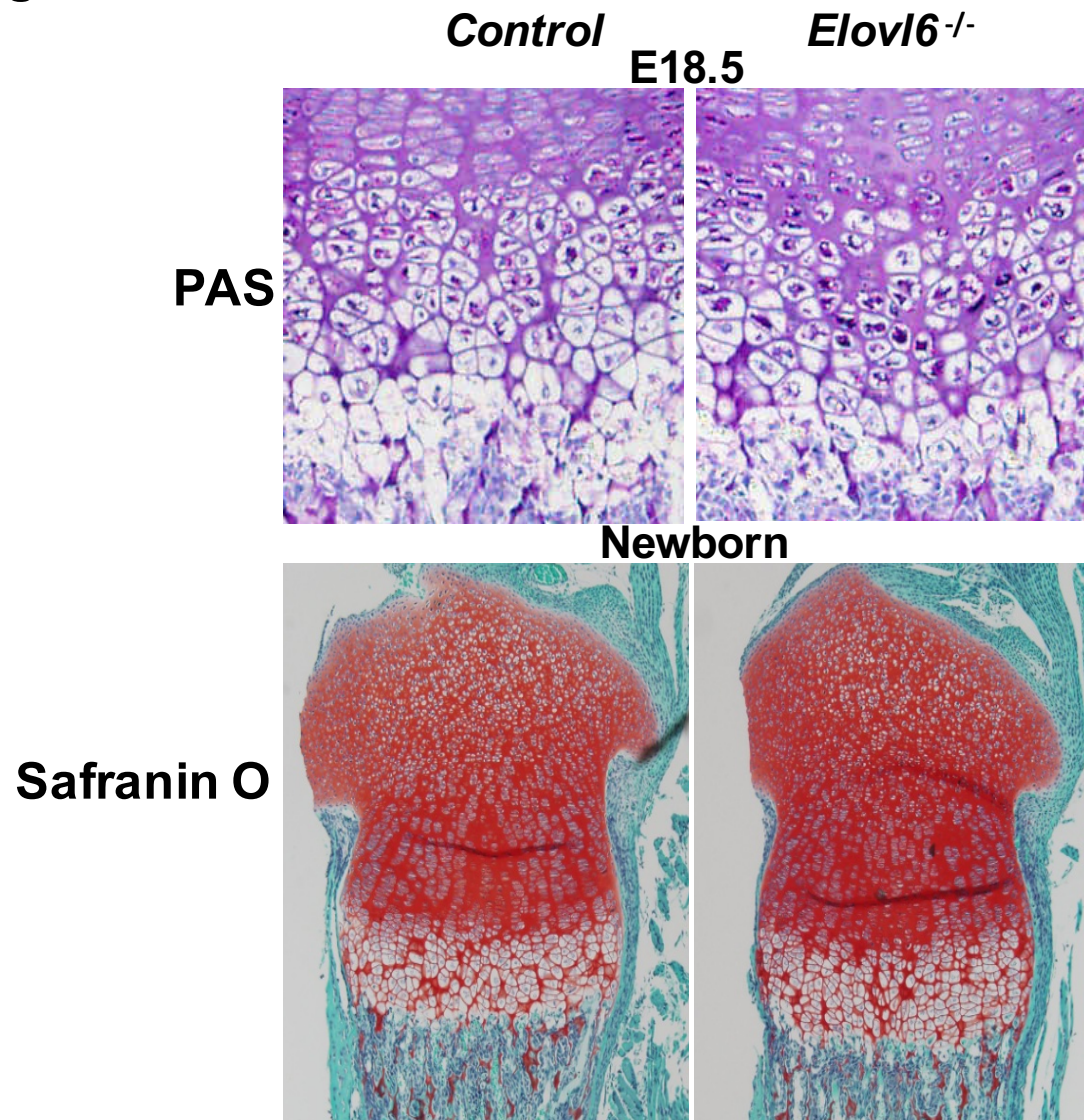

Supplement: S2 Fig — Cartilage polysaccharides and proteoglycans were detected by (A) PAS and (B) safranin O staining, respectively, between control and Elovl6-/- mice. (PDF) [file pone.0159375.s002.pdf]

S3 Figure

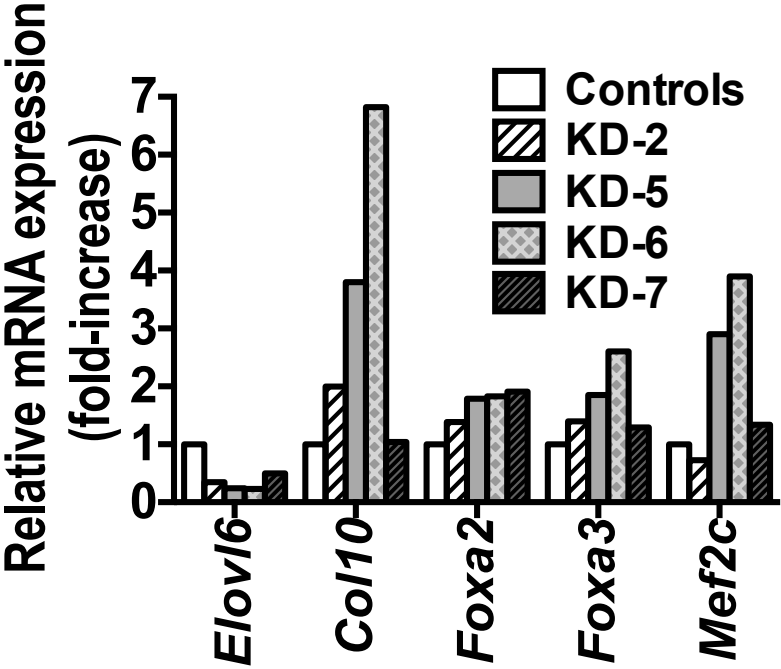

Supplement: S3 Fig — Four clonal ATDC5 cell lines (KD-2, 5, 6, and 7) were established using shElovl6. Cells were incubated in a differentiation medium for 7 days and expression of Col10α1, Foxa2, Foxa3, and Mef2c were assessed by real-time qPCR. Based on this result, we used two cell lines, KD-5 and KD-6, whose Elovl6 expression were reduced to about 20% of that of control cells, for our experiments. (PDF) [file pone.0159375.s003.pdf]
